# Olfactory rehabilitation and olfactory bulb volume changes in patients after total laryngectomy: a prospective randomized study

**DOI:** 10.1016/j.bjorl.2021.02.013

**Published:** 2021-03-20

**Authors:** Defne Gürbüz, Mustafa Caner Kesimli, Ahmet Mert Bilgili, Hacı Ömer Durmaz

**Affiliations:** a University of Health Sciences, Prof. Dr. Cemil Taşçıoğlu City Hospital, Department of Radiology, İstanbul, Turkey; b Istinye University, School of Medicine, Department of Otolaryngology, Head and Neck Surgery, İstanbul, Turkey; c Cyprus International University, Medical Faculty, Department of Otolaryngology, Head and Neck Surgery, Lefkoşe, Cyprus; d Avicenna Private Hospital, Otorhinolaryngology Department, İstanbul, Turkey

**Keywords:** Olfactory, Laryngectomy, Orthonasal, Rehabilitation, Training

## Abstract

**Introduction:**

After total laryngectomy, decreased olfactory function and olfactory bulb volume shrinkage have been reported to occur due to olfactory deprivation caused by nasal airflow interruption. There is evidence that the olfactory system can be modulated by repeated exposure to odors in a procedure called olfactory training. However, it is not known whether any recovery of the lost olfactory bulb volume is possible by eliminating olfactory deprivation via olfactory rehabilitation long after laryngectomy.

**Objective:**

This study examined the recovery of olfactory function and the change in olfactory bulb volume via long-term olfactory rehabilitation after total laryngectomy.

**Methods:**

Possible causes of olfactory dysfunction in the study participants were evaluated by collecting detailed anamnesis. As olfactory tests, orthonasal butanol threshold and odor discrimination tests were performed. Three-dimensional olfactory bulb volumes were calculated using manual segmentation on T2-weighted coronal magnetic resonance images. In olfactory rehabilitation, four different odors were applied to all patients orthonasally, using a larynx bypass technique for 30 min per day for 6 months. Olfactory tests were performed before the rehabilitation and after 6 months of rehabilitation, and olfactory bulb volume measurements were performed using magnetic resonance images.

**Results:**

Eleven patients diagnosed with advanced laryngeal cancer who underwent total laryngectomy and postoperative radiotherapy with a follow-up of 5–10 years were included in the study. All patients were male, and the mean age was 58.18 ± 4.17 years. In total laryngectomized patients, the olfactory bulb volumes measured on magnetic resonance images were 42.25 ± 12.8 mm^3^ before and 55.5 ± 11.22 mm^3^ after rehabilitation, and this increase was highly significant. Olfactory test scores were 2.3 ± 1.27 before and 4.39 ± 0.86 after rehabilitation, and this increase was also highly significant.

**Conclusion:**

As a result of the olfactory rehabilitation applied by providing orthonasal air flow, the olfactory function lost after total laryngectomy was improved considerably, and the olfactory bulb volume was significantly increased. The increase in olfactory bulb volume in total laryngectomy patients via olfactory rehabilitation to eliminate olfactory deprivation due to nasal airflow interruption was demonstrated for the first time in this prospective longitudinal study.

## Introduction

Olfactory loss not only negatively affects quality of life but may even jeopardize the patient’s personal safety because of the inability to detect smoke or other dangerous olfactory signals.^1^

The olfactory nerve is a nerve pertaining to sensory function of smell (olfaction) alone. The olfactory system consists of the epithelium, bulb, and tracts associated with the cortical olfactory area, also known as the rhinencephalon. The olfactory bulb (OB) consists of the sensory afferents of the olfactory receptor cells located in the olfactory neuroepithelium. The OB is thought to be the most important station of the olfactory pathways connecting the peripheral and cortical structures. The OB reflects the level of afferent neural activity and maintains a degree of plasticity throughout adult life.^2^

After total laryngectomy, air flow does not reach the olfactory mucosa due to interrupted nasal airflow; as a result, the olfactory center is deprived of olfactory stimuli. Frequently, this deprivation soon leads to severe hyposmia and anosmia. Despite being reported in laryngectomized patients, this olfactory loss has been widely overlooked in previous studies.^1^, ^2^, ^3^ As a result of olfactory stimulus deprivation, the OB volume diminishes due to decreased cell numbers over time.^4^ Interrupted nasal breathing as a result of tracheostomy after laryngectomy leads to significant olfactory loss in patients. Significantly impaired olfactory ability has been observed in laryngectomized patients.^5^, ^6^

Magnetic resonance imaging (MRI) is a reliable and ideal technique for OB volume measurements. The OB volume also reflects the functional status of this structure in the human olfactory system due to its plasticity. Regarding OB volume measurements, post-traumatic olfactory dysfunction and neurodegenerative diseases have been evaluated in total laryngectomized patients and individuals with normal olfaction.^7^, ^8^, ^9^ Veyseller et al. demonstrated in both cross-sectional and longitudinal studies that olfactory dysfunction and OB volume shrinkage occur in laryngectomy patients.^5^, ^6^

As yet, no effective method is universally accepted for the treatment of olfactory dysfunction. Olfactory rehabilitation through olfactory training consists of modulating the systemic regeneration process by repeatedly exposing the olfactory system to particular odors.^10^

Significant improvements in olfactory test results after olfactory training in patients with post-traumatic and post-infectious olfactory dysfunction have been reported.^11^ The training is performed once a day using four odors: phenylethyl alcohol (rose), eucalyptol (eucalyptus), citronellal (lemon), and eugenol (clove).^12^, ^13^ There are also studies on the improvement of olfactory function in laryngectomy patients via olfactory rehabilitation.^1^, ^14^, ^15^

This study was conducted prospectively and longitudinally in patients after laryngectomy to demonstrate improvements in olfactory function and changes in OB volumes via olfactory rehabilitation achieved by restoration of nasal air flow.

## Methods

This prospective study was conducted on patients admitted to the Ear, Nose and Throat (ENT) and Head and Neck Surgery Clinic with the assistance of the Radiology Clinic. The study was carried out in accordance with the Declaration of Helsinki (WMA 1997). Approval was obtained from the ethics committee, and the participants gave written consent after being informed about the study. (Approval number of the ethics committee: (2017-KAEK-120)/2/2020.G-068).

Patients with a history of nasal polyposis, neurological or psychiatric diseases, head trauma or nasal surgery that may cause olfactory impairment before laryngectomy were excluded. Routine ENT examinations, paranasal sinus computed tomography, orthonasal olfactory tests, and cranial MRI were performed for OB volume measurements in all patients. Cranial MRI and orthonasal olfactory tests were conducted before and 6-months after laryngectomy. In addition, a complete neurological examination and Mini-Mental evaluation tests, as well as cranial MRI, were performed to exclude possible cognitive disorders or neurodegenerative diseases.

The orthonasal olfactory test devised by the Connecticut Chemosensory Clinical Research Center (CCCRC) was used.^16^, ^17^ The possible score ranges obtained from the CCCRC orthonasal tests are shown in Table 1.

**Table 1:** CCCRC score ranges.

	Score ranges
Anosmia	0–1.75
Severe hyposmia	2–3.75
Moderate hyposmia	4–4.75
Mild hyposmia	5–5.75
Normosmia	6–7

**Figure 1:**
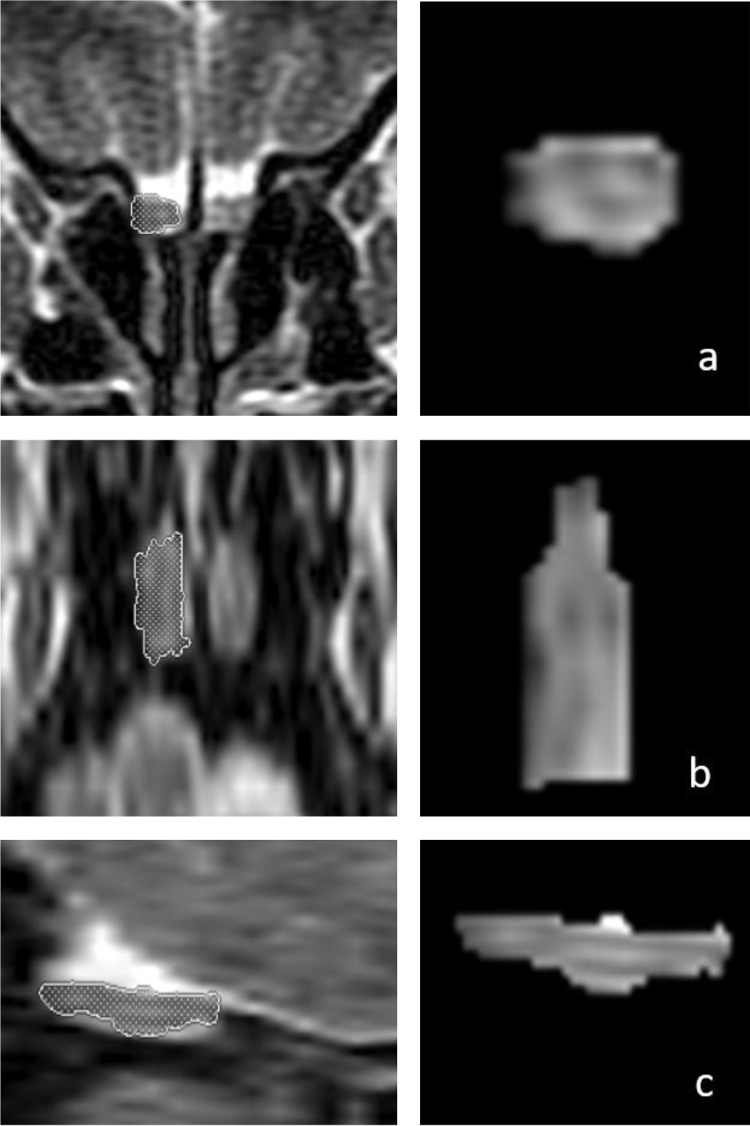
Three-dimensional measurements of the right olfactory bulb (OB) volume in a 66-year-old male patient on T2-weighted coronal (a, first row), axial (b, middle row), and sagittal (c, bottom row) magnetic resonance images. The patient’s right OB volume was 76 mm^3^ after olfactory rehabilitation.

**Figure 2:**
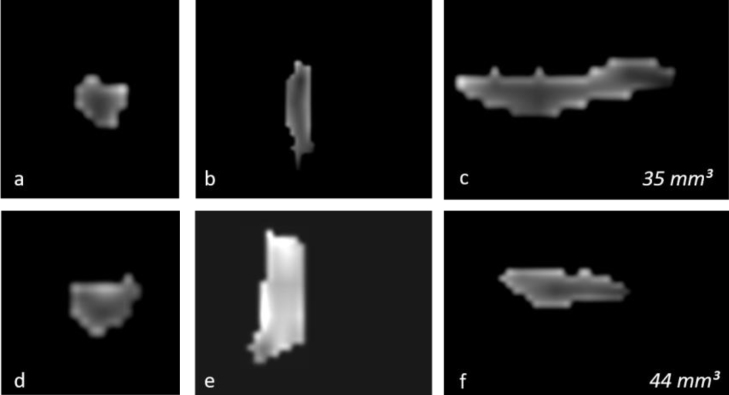
Changes in the three-dimensional volume measurement of the left OB in a total laryngectomized patient on magnetic resonance images, before olfactory rehabilitation (a–c, upper row) and after olfactory rehabilitation (d–f, lower row), as coronal (a, d), axial (b, e), and sagittal (c, f) images, respectively. The left OB volume was 35 mm^3^ and 44 mm^3^ before and after olfactory rehabilitation.

OB volume measurements were performed by MRI images (Fig. 1) using the 1.5T General Electric Signa Excite MRI device by three-dimensional evaluation on coronal T2-weighted Turbo Spin Echo (TSE) sections using a manual segmentation method. The sections had a slice thickness of 2 mm without intersection gaps (gap = 0). All measurements were performed by the same experienced radiologist, separately from both sides, without knowledge of whether the images were obtained before or after treatment. OB volume measurements were calculated in mm^3^ (Figure 1, Figure 2).^11^, ^12^

Patients with post-traumatic parenchymal or meningeal hemosiderin accumulation in brain tissues on T2-weighted Gradient Echo Sequence (GRE) MRI sections were excluded from the study. In addition, conventional cranial MRI sequences were screened for other organic disorders of the brain, and individuals with any pathology were excluded from the study.

For olfactory rehabilitation, air flow was provided to the nasal olfactory mucosa, and access of odor particles to the olfactory region was achieved using a larynx bypass technique. During olfactory rehabilitation, the training was performed for 30 min a day for 6 months using four odors: phenylethyl alcohol (rose), eucalyptol (eucalyptus), citronellal (lemon), and eugenol (clove).

In this section, we perform statistical analysis to investigate if the rehabilitation helps counteract the reduction in CCCRC scores and OB volumes due to laryngectomy (i.e., are the CCCRC scores and OB volumes higher after 6 months of postoperative rehabilitation compared to right after the laryngectomy?). We consider that the results with a *p*-value less than 0.05 are statistically significant.

Descriptive statistics are presented in the Table 2. The majority of the patients are middle-aged (range: 52–66), and the ANCOVA analysis reveals that “age” does not have a statistically significant effect on the difference in CCCRC scores and OB volumes pre/post rehabilitation, and thus can be safely eliminated from further analysis.

**Table 2:** Descriptive statistics.

	Group 1 (pre rehab, n = 11)				Group 2 (post rehab, n = 11)			
Variables	Mean	SD	Median	Range	Mean	SD	Median	Range
Age (yr)	58.18	4.17	58	(52–66)	58.18	4.17	58	(52–66)
CCCRC	2.3	1.27	1.75	(0.5–4.5)	4.39	0.86	4.5	(2–5.25)
OB	42.5	12.8	43	(28.5–63)	55.5	11.22	54.5	(38.5–77.5)

OB, olfactory bulb volume; CCCRC, Connecticut Chemosensory Clinical Research Center test score.

**Figure 3:**
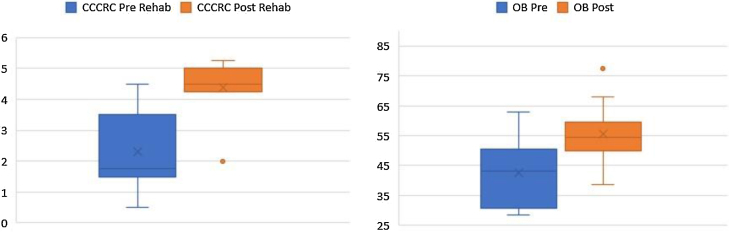
Box plots for CCCRC scores and OB volumes pre/post rehabilitation.

According to the box plots in Fig. 1, the CCCRC scores and OB volumes are noticeably higher as a result of the rehabilitation, in line with our prior belief that the rehabilitation helps increase CCCRC scores and OB volumes. The Wilcoxon test for paired samples also confirms that the post-rehabilitation CCCRC scores and OB volumes are higher than the pre-rehabilitation values measured 6-months apart (highly statistically significant with *p*-values of 0.001) (Fig. 3).

## Results

A total of 11 patients diagnosed with advanced laryngeal cancer were included. All patients were male. The youngest and oldest patients were 52 and 66 years old, with a mean age of 58.18 ± 4.17 years. All patients had a history of smoking — mean 25 (range 10–40) cigarettes/day.

In all 11 laryngectomized patients, the mean OB volume before rehabilitation was 42.5 (with a standard deviation of 12.8 and range 28.5–62.5) mm^3^ (Table 2), while the mean OB volume measured after 6 months of olfactory rehabilitation was 55.5 (with a standard deviation of 11.22 and range 39.5–77.5) mm^3^. The OB volume was significantly larger after than before olfactory rehabilitation (*p* < 0.001) (Fig. 1).

CCCRC mean scores (butanol threshold and odor identification scores) were 2.3 ± 1.27 (range 0.50–3.25) and 4.39 ± 0.86 (range 2.50–5.25) (out of 7) before and after the 6-month olfactory rehabilitation, respectively. The increase in the score after the rehabilitation (at the end of 6-months) was statistically significant (*p* < 0.001) (Fig. 1). According to the CCCRC scoring system, the score of 2.3 in patients before rehabilitation indicated severe hyposmia, while the score of 4.39 after rehabilitation demonstrated moderate hyposmia.

According to the CCCRC scores before rehabilitation, one patient was moderately hyposmic, three were severely hyposmic, and six were anosmic. After rehabilitation, three patients were mildly hyposmic, five were moderately hyposmic, and two were severely hyposmic (Table 3).

**Table 3:** Patient distribution among the CCCRC score categories.

Category	Before rehabilitation	6-months after rehabilitation
Normal	0	0
Mild hyposmia	0	3
Moderate hyposmia	1	5
Severe hyposmia	3	2
Anosmia	6	0
Total	10	10

## Discussion

Due to tracheostomy performed after total laryngectomy, the nasal airway is interrupted, and the neuroepithelium specialized for olfaction is deprived of odor particles.^6^ Two mechanisms potentially explaining olfactory loss after laryngectomy are interruption of nasal airflow after laryngectomy and impairment of neurosensory feedback mechanisms after multiple peripheral nerve injuries during surgery.^5^ The OB displays plasticity throughout adult life, based on two major neurobiological mechanisms; one is related to the continuity of neurogenesis from the subventricular zone, and the other is related to the continuity of synaptogenesis.^18^

A reduction in OB volumes resulting in hypoplasia has been shown to be the most important effect of olfactory stimuli deprivation in animals.^4^ The continuity in the OB neuroblast flow from the subventricular zone in humans is described in the CNS (Central Nervous System).^18^

Bulbar neuroplasticity is associated with input from olfactory receptor neurons.^19^ The continuity of neurogenesis, which is sensitive to environmental factors and stimuli, results in neural recruitment, and sensitivity improves OB volume.^19^

Shehata et al. demonstrated that in patients with chronic rhinosinusitis with nasal polyposis, postoperative improvement in olfactory function and an increase in OB volumes occurred 6 months after removal of the nasal blockade.^20^

Although pulmonary and vocal problems after total laryngectomy have been widely covered in the literature, there are few studies on the deterioration of olfactory function.^1^ OB volumes measured postoperatively in laryngectomized patients were reported to be lower than those in a similar age control group.^5^, ^6^ Atrophy of the olfactory neuroepithelium or the OB may play a role in the emergence of olfactory problems in patients after laryngectomy.^6^, ^18^, ^19^, ^21^

The pathophysiology of olfactory reduction in laryngectomized patients is not fully understood, and several theories have been proposed. The interruption of nasopulmonary air flow in laryngectomized patients automatically interrupts the passive sniffing mechanism. Miani et al. suggested that degeneration of the olfactory epithelium may result from atrophy, inflammation, or disuse. Laryngectomy patients show severe degeneration of the olfactory epithelium.^22^

In our study, although more than 5 years had passed since laryngectomy and olfactory loss, the patients responded to olfactory rehabilitation, and both odor perception and OB volumes increased. This supports the fact that, after its activation, the olfactory system can regain its former functions and structure due to its plasticity.

Our study is important in that it showed increases in both CCCRC scores and OB volumes as a result of olfactory rehabilitation after laryngectomy.

## Conclusion

Olfactory function can recover even after several years after laryngectomy using olfactory rehabilitation, and consequently, OB volumes can increase.

## Conflicts of interest

The authors declare no conflicts of interest.
